# Long-term bone remodeling of maxillary anterior teeth with post-treatment alveolar bone defect in adult patients with maxillary protrusion: a prospective follow-up study

**DOI:** 10.1186/s40510-023-00489-w

**Published:** 2023-11-06

**Authors:** Runzhi Guo, Linwei Li, Yifan Lin, Yiping Huang, Jian Liu, Mengqiao Pan, Li Xu, Weiran Li

**Affiliations:** 1 Department of Orthodontics, Peking University School and Hospital of Stomatology, 22 Zhongguancun Avenue South, Haidian District, Beijing, 100081 People’s Republic of China; 2 Division of Pediatric Dentistry and Orthodontics, Faculty of Dentistry, The University of Hong Kong, Hong Kong, SAR China; 3 Department of Periodontics, Peking University School and Hospital of Stomatology, Beijing, China

**Keywords:** Alveolar bone defect, Maxillary anterior teeth retraction, Long-term bone remodeling, Stability

## Abstract

**Background:**

Alveolar bone defects, particularly palatal bone dehiscence (PBD) and labial bone fenestration (LBF), occur frequently as a result of retraction of the maxillary anterior teeth. The study aims to explore the long-term bone remodeling of maxillary anterior teeth in adult patients with post-orthodontic treatment PBD and LBF.

**Materials and methods:**

The study includes 24 adult patients with maxillary protrusion (8 males, 16 females) who were treated with extraction of four first premolars and had alveolar bone defects (PBD or LBF) in maxillary anterior teeth following orthodontic treatment. Cone-beam computed tomography imaging measurements were obtained before (T1), after (T2) orthodontic treatment, and after at least 1-year removable thermoplastic retainer retention (T3). The maxillary anterior teeth with PBD or LBF at T2 were divided into the PBD or LBF groups, respectively. The labial and palatal alveolar bone height (ABH), alveolar bone thickness (ABT), and movement of maxillary anterior teeth were measured during retraction (T2–T1) and retention (T3–T2) periods.

**Results:**

The incidence of PBD and LBF in maxillary anterior teeth significantly increased after orthodontic treatment and decreased during the retention period. In the PBD group, the palatal ABH of all maxillary anterior teeth significantly increased from T1 to T2 but decreased from T2 to T3. The ABT of the maxillary central incisor and canine significantly increased on the palatal side and decreased on the labial side during the retention period. In the LBF group, the labial ABT of the maxillary central incisor at the apical level showed a significant decrease from T1 to T2, followed by an increase from T2 to T3. In both groups, the maxillary central incisor showed significant labial movement, with a relative intrusion during the retention period.

**Conclusion:**

For adult patients with maxillary protrusion, the alveolar bone defect of maxillary anterior teeth caused by orthodontic retraction significantly improved during the retention period, indicating good long-term bone remodeling. Our findings suggest that a combination of spontaneous reorientation of maxillary anterior teeth and bone remodeling contributed to alveolar bone covering in these patients.

## Background

Maxillary protrusion is common in patients with Class I and Class II malocclusion, and particularly in the Asian population [1, 2]. The extraction of maxillary premolars and subsequent retraction of maxillary anterior teeth are commonly performed to achieve an esthetic profile and better occlusion in these patients. Recently, the widespread use of mini-implants has remarkably increased the extent of retraction of the maxillary anterior teeth, which poses significant risk of alveolar bone defects in the maxillary anterior teeth following orthodontic treatment [3]. The alveolar bone in maxillary anterior teeth is always thin in maxillary protrusion patients [4, 5]. Evangelista et al. [6] reported that the incidence of alveolar bone defects (dehiscence and fenestration) in non-orthodontic patients with Class I and Class II Division I malocclusion was 36.51% and 51.09%, respectively. Hence, these patients have a high risk of alveolar bone defects in maxillary anterior teeth during orthodontic treatment.

During maxillary anterior teeth retraction, the tooth movement commonly exceeds the bone apposition, in which the ratio of bone remodeling to anterior teeth retraction is less than 1:1, particularly on the palatal side. Once the degree of retraction becomes excessive, such that it exceeds the palatal alveolar bone boundary, palatal bone dehiscence (PBD) in the cervical region could occur [7]. In our previous systematic review, we found that the palatal alveolar bone height and thickness both significantly decreased during maxillary anterior teeth retraction [8]. Other than PBD, if the torque control of maxillary anterior teeth is insufficient during retraction, the teeth tend to incline lingually; thus, labial bone fenestration (LBF) in the apical region could be present. With the common use of cone-beam computed tomography in orthodontic fields, the three-dimensional morphological change in the root and alveolar bone during anterior teeth retraction can be easily detected [5]. Several CBCT studies have reported the frequent occurrence of PBD and LBF in maxillary anterior teeth during retraction [7, 9–12]. Hence, the periodontal safety of retraction treatment should be an essential concern for both orthodontists and periodontists.

The issue of bone remodeling after tooth movement has been extensively discussed. Various techniques, including orthodontic extrusion and orthodontic implant site switching, have been proposed to preserve alveolar bone volume and facilitate subsequent prosthodontic or implant treatment [13]. However, questions still remain regarding whether bone remodeling can take place during the retention phase, especially when alveolar bone defects occur in the maxillary anterior teeth after orthodontic retraction. At present, there is limited research investigating the long-term changes in alveolar bone in maxillary anterior teeth, and the question of alveolar bone remodeling during the retention phase remains [14–16]. In a study by Remmelink et al. [16], the cortical plate after orthodontic treatment did not recover when the root penetrated the palatal cortical plate. By contrast, a CBCT study found that loss of palatal alveolar bone in adolescent patients, who tend to have greater bone remodeling capacity, could recover during the retention phase [15]. For adult patients with limited bone remodeling capacity, long-term bone remodeling of maxillary anterior teeth during the retention phase has not been investigated. Hence, we explored the long-term bone remodeling capability of maxillary anterior teeth in adult orthodontic patients with post-treatment alveolar bone defects.

## Materials and methods

### Participants

This study was approved by the Peking University School and Hospital of Stomatology Ethics Committee (PKUSSIRB-202168141). All patients provided written informed consent before participating. The sample size was calculated by Power Analysis and Sample Size software (PASS 2000, NCSS, Kaysville, UT, USA) based on a previous study that reported a palatal ABH of the maxillary central incisor of 2.43 ± 1.76 mm after orthodontic treatment and 1.23 ± 0.63 mm after 2 years of retention [15]. Twenty-one patients were required to meet the clinical difference, given an analysis assuming 80% power at the 5% significance level (two-sided). In total, 24 adult patients from the Department of Orthodontics, Peking University School and Hospital of Stomatology, who had alveolar bone defect in maxillary anterior teeth after orthodontic treatment, were recalled after at least 1 year of retention and included in this study.

The inclusion criteria were as follows: age > 18 years, maxillary protrusion (Class I or Class II molar relationship, 2° < ANB angle < 8°, U1-NA angle > 22.8° [17], upper lips positioned in advance of the E-line), extraction of four first premolars, undergone maxillary anterior teeth retraction during orthodontic treatment, at least one maxillary anterior tooth diagnosed with PBD or LBF after orthodontic treatment, available CBCT scans before and after orthodontic treatment, and wearing a removable thermoplastic retainer during retention period. The exclusion criteria were as follows: missing maxillary anterior teeth, maxillary anterior teeth with root canal treatment and crown, significant reopening of the extraction site or crowding during the retention period, periodontal inflammation, systemic disease, and smoker. All included patients were treated by three experienced orthodontists (RZ Guo, YP Huang and WR Li) using pre-adjusted MBT brackets with a 0.022″ × 0.028″ slot (3 M Unitek, Monrovia, CA, USA). Sliding mechanics with 0.019″ × 0.025″ stainless steel was used to retract the maxillary anterior teeth.

### Cone beam computed tomography imaging

To analyze the long-term periodontal safety of maxillary anterior teeth with post-treatment alveolar bone defect, all patients were recalled to take a CBCT scan after at least 1 year retention (T3). The pre- (T1) and post-treatment (T2) CBCT were obtained retrospectively. All CBCT scans were taken using a NewTom Scanner (Marburg, Germany) under the following conditions: axial slice thickness, 0.25 mm; field of view, 16 cm × 16 cm; and scan time, 15 s. The CBCT raw data were exported into DICOM format and imported into Dolphin 3D Imaging software (version 11.8, Dolphin Imaging and Management Solutions, Chatsworth, CA, USA) for further analysis.

All CBCT images were standardized and oriented as follows: the horizontal plane was defined as the Frankfort horizontal plane (Or-Po); the mid-sagittal plane was defined as the plane connecting the nasion, anterior nasal spine, and posterior nasal spine (Na-ANS-PNS). The maxillary region was selected for voxel-based superimposition as described previously [18]. The CBCT images at T1 and T2 were superimposed to analyze the teeth movement and alveolar bone change before and after orthodontic treatment, and CBCT images at T2 and T3 were superimposed to analyze the teeth movement and alveolar bone change during the retention period.

### Measurement of teeth movement

To analyze the maxillary anterior teeth movement, a 3D coordinate system in the superimposition model was established, as described previously [18]. The Na-ANS-PNS plane (sagittal plane), Frankfort horizontal plane (axial plane), and the plane perpendicular to these two planes (coronal plane), were selected as the X, Y, and Z planes, respectively. The origin point (0, 0, 0) was the PNS point. The midpoint of the incisal edge, cusp tip of the canine, and the root apex of maxillary anterior teeth were manually positioned and selected as landmarks. The coordinates of each landmark were exported into Microsoft Excel 2013 (Microsoft Corp., Redmond, WA, USA) and analyzed using MathType software (ver. 5.0, Design Science, Long Beach, CA, USA). The amount of retraction and relapse of the maxillary anterior teeth, at the crown and root apex levels, were measured as the distances between landmarks at T1 and T2, and at T2 and T3, respectively.

### Measurement of alveolar bone

**Fig. 1:**
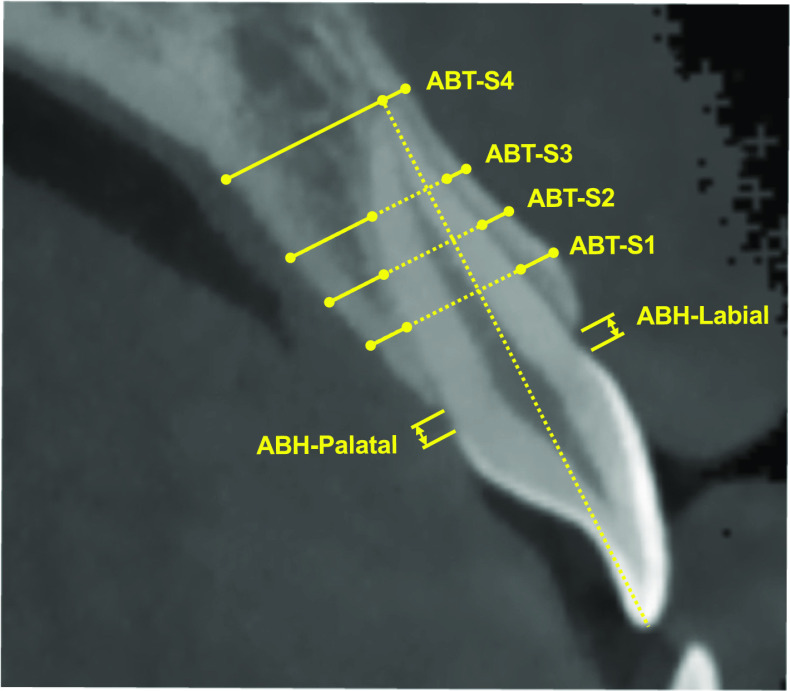
Schematic of alveolar bone height (ABH) and alveolar bone thickness (ABT) in maxillary anterior teeth

As shown in Fig. 1, the alveolar bone height (ABH) and alveolar bone thickness (ABT) were measured to detect bone defect and evaluate bone remodeling. For each maxillary anterior tooth, the long axis (the line connecting the midpoint of the incisal edge/the cusp tip and root apex) was selected as the reference line. The distance between the alveolar bone crest and cemento-enamel junction (CEJ), parallel to the long axis, was measured as ABH. ABT was measured as the distance between the cortical plate and root surface, perpendicular to the long axis, at S1 (4 mm from CEJ), S2 (6 mm from CEJ), S3 (8 mm from CEJ), and S4 (root apex) levels. According to the study by Davies et al. [19], dehiscence was diagnosed when the labial or palatal alveolar bone crest was at least 4 mm apical to inter-proximal bone margin. Fenestration was diagnosed as a localized defect of alveolar bone exposing the root at least 3 consecutive sections, without involving the alveolar bone margin. The incidence of PBD and LBF at T1, T2, and T3 were recorded. To further analyze the bone remodeling of maxillary anterior teeth with post-treatment alveolar bone defect, the maxillary anterior teeth with PBD and LBF at T2 were divided into the PBD and LBF groups, separately. All measurements of maxillary anterior teeth were performed at the labial and palatal sides.

### Statistical analysis

All CBCT measurements were conducted by one experienced orthodontist (LW Li). To assess measurement reliability, five patients were selected at random. The CBCT measurements of these five patients were repeated at 2-week intervals. The Dahlberg formula was used to measure the method error. The Shapiro–Wilk test was performed to test the normality of measurement distributions. When measurements were normally distributed, a repeated-measures analysis of variance and post hoc least significant difference tests were used to evaluate differences among the three time points. Paired t tests were used to evaluate the teeth movement in each group from T1 to T2 and T2 to T3. If the measurement distribution was not normally distributed, the Wilcoxon signed-rank test was used. The generalized estimating equation was used to compare the difference in incidence among groups. All statistical analyses were performed using SPSS software (version 26; SPSS, Chicago, IL, USA) with a statistical significance level of 0.05.

## Results

The intra-class correlation coefficients of the CBCT measurements were all higher than 0.90, indicating good reliability. The method error ranged from 0.11 to 0.42 mm for teeth movement measurements and 0.09 to 0.28 mm for bone measurements. The patients’ descriptive statistics are shown in Table 1.

**Table 1:** The demographic information of including patients

Measurements	Mean ± SD	
*Demographic*		
Age (years)	24.86 ± 4.10	
Sex (male / female)	8/16	
Angle classification (Class I / Class II)	14/10	
Skeletal classification (Class I / Class II)	7/17	
Overbite at T1 (mm)	1.64 ± 1.13	
Overjet at T1 (mm)	3.63 ± 2.00	
Treatment duration (years)	2.89 ± 0.58	
Retention duration (years)	1.52 ± 0.50	
*Pre-treatment Cephalometric analysis*		
SNA (°)	83.31 ± 2.83	
SNB (°)	77.61 ± 2.76	
ANB (°)	5.71 ± 1.12	
SN-MP (°)	38.50 ± 2.67	
U1-SN (°)	112.71 ± 8.55	
U1-NA (°)	24.22 ± 4.45	

^*^P < 0.05, ^**^P < 0.01

### Incidence of alveolar bone defects in maxillary anterior teeth

The incidence of alveolar bone defect is shown in Table 2. Among 144 maxillary anterior teeth, there was no maxillary anterior teeth with PBD at T1. The incidence of PBD in maxillary anterior teeth at T2 was 67.36% and was significantly lower at T3 (11.11%). After orthodontic treatment, the maxillary lateral incisors showed the highest incidence of PBD (77.08%), followed by maxillary central incisors (75.00%) and maxillary canine (50.00%). As for LBF, the incidence in maxillary anterior teeth was 20.83% at T1, 24.31% at T2, and 10.42% at T3, respectively. Among the maxillary anterior teeth, the incidence of LBF was highest in the maxillary canine (37.50% at T1, 41.67% at T2, and 20.83% at T3).

**Table 2:** The incidence of alveolar bone defect of maxillary anterior teeth

Incidence rate (%)	T1	T2	T3	Overall	T1 vs T2	T2 vs T3	T1 vs T3
				*p*	*p*	*p*	*p*
*Maxillary central incisor (n* = *48)*							
PBD	0	75.00	2.08	< 0.001^***^	< 0.001^***^	< 0.001^***^	< 0.001^***^
LBF	4.17	10.42	0	0.017^*^	0.250	0.018^*^	0.149
*Maxillary lateral incisor (n* = *48)*							
PBD	0	77.08	10.42	< 0.001^***^	< 0.001^***^	< 0.001^***^	0.018^*^
LBF	20.83	20.83	10.42	0.024^*^	1.000	0.018^*^	0.049^*^
*Maxillary canine (n* = *48)*							
PBD	0	50.00	20.83	< 0.001^***^	< 0.001^***^	< 0.001^**^	< 0.001^***^
LBF	37.50	41.67	20.83	0.020^*^	0.562	0.012^*^	0.014^*^
*All maxillary anterior teeth (n* = *144)*							
PBD	0	67.36	11.11	< 0.001^***^	< 0.001^***^	< 0.001^***^	< 0.001^***^
LBF	20.83	24.31	10.42	< 0.001^***^	0.334	< 0.001^***^	0.001^**^

^*^P < 0.05; ^**^ *P* < 0.01; ^***^ *P* < 0.001

### Bone remodeling and tooth movement of maxillary anterior teeth in the PBD group

There were 97 maxillary anterior teeth with PBD at T2 (36 maxillary central incisors, 37 maxillary lateral incisors, and 24 maxillary canines) in the PBD group. As shown in Table 3, the palatal ABH of all maxillary anterior teeth significantly increased after orthodontic treatment and decreased after at least 1 year of retention (central incisor: 1.028 ± 0.407 mm at T1, 6.735 ± 1.729 mm at T2, 2.169 ± 0.993 mm at T3; lateral incisor: 1.216 ± 0.497 mm at T1, 8.442 ± 11.477 mm at T2, 2.555 ± 1.472 mm at T3; canine: 1.225 ± 0.464 mm at T1, 5.790 ± 1.654 mm at T2, 3.164 ± 1.477 mm at T3), indicating a recovery of bone dehiscence during the retention period (T3–T2). There were no significant differences in the change in the labial ABH of maxillary anterior teeth among the three time points. As for bone thickness, the ABT of the maxillary central incisor and canine significantly increased on the palatal side and decreased on the labial side during the retention period, while only a significant increase in palatal ABT at the S1 level was observed in maxillary lateral incisors at T3.

**Table 3:** The alveolar bone thickness and height of maxillary anterior teeth in PBD group

	T1		T2		T3		Overall	T1 vs T2	T2 vs T3	T1 vs T3
	Mean	SD	Mean	SD	Mean	SD	*p*	*p*	*p*	*p*
*Maxillary central incisor*										
Palatal-ABT-S1 (mm)	1.786	0.656	0.290	0.323	0.950	0.379	< 0.001^***^	< 0.001^***^	< 0.001^***^	< 0.001^***^
Palatal-ABT-S2 (mm)	2.464	1.128	0.622	0.946	1.388	0.615	< 0.001^***^	< 0.001^***^	< 0.001^***^	< 0.001^***^
Palatal-ABT-S3 (mm)	3.397	1.705	1.637	1.772	2.248	1.223	< 0.001^***^	< 0.001^***^	< 0.001^***^	0.001^**^
Palatal-ABT-S4 (mm)	7.231	2.338	5.118	2.630	5.607	2.242	< 0.001^***^	< 0.001^***^	0.001^**^	0.001^**^
Labial-ABT-S1 (mm)	1.169	0.353	1.680	0.494	1.372	0.342	< 0.001^***^	< 0.001^***^	< 0.001^***^	0.011^*^
Labial-ABT-S2 (mm)	0.984	0.471	1.840	0.647	1.358	0.482	< 0.001^***^	< 0.001^***^	< 0.001^***^	0.001^**^
Labial-ABT-S3 (mm)	0.827	0.477	1.928	0.892	1.402	0.655	< 0.001^***^	< 0.001^***^	< 0.001^***^	< 0.001^***^
Labial-ABT-S4 (mm)	1.442	0.552	3.372	1.507	2.649	1.202	< 0.001^***^	< 0.001^***^	< 0.001^***^	< 0.001^***^
Palatal-ABH (mm)	1.028	0.407	6.735	1.729	2.169	0.993	< 0.001^***^	< 0.001^***^	< 0.001^***^	< 0.001^***^
Labial-ABH (mm)	1.758	0.639	1.812	0.783	1.844	0.645	0.451	0.494	0.607	0.192
*Maxillary lateral incisor*										
Palatal-ABT-S1 (mm)	1.404	0.427	0.260	0.326	0.529	0.271	< 0.001^***^	< 0.001^***^	0.001^**^	< 0.001^***^
Palatal-ABT-S2 (mm)	1.925	0.720	0.740	1.690	0.937	0.420	< 0.001^***^	< 0.001^***^	0.451	< 0.001^***^
Palatal-ABT-S3 (mm)	2.681	0.910	1.875	4.284	1.456	0.815	0.134	0.270	0.539	< 0.001^***^
Palatal-ABT-S4 (mm)	5.910	1.486	5.773	10.707	4.228	1.351	0.376	0.941	0.381	< 0.001^***^
Labial-ABT-S1 (mm)	0.991	0.458	2.234	5.510	1.164	0.481	0.204	0.180	0.238	0.006^**^
Labial-ABT-S2 (mm)	0.621	0.588	2.236	6.735	0.921	0.626	0.174	0.142	0.227	< 0.001^***^
Labial-ABT-S3 (mm)	0.389	0.544	1.923	5.193	0.838	0.591	0.102	0.068	0.189	< 0.001^***^
Labial-ABT-S4 (mm)	1.393	0.593	3.219	4.823	2.131	0.845	0.054	0.029^*^	0.181	< 0.001^***^
Palatal-ABH (mm)	1.216	0.497	8.422	11.477	2.555	1.472	0.002^**^	0.001^**^	0.005^**^	< 0.001^***^
Labial-ABH (mm)	1.847	0.774	2.806	4.858	2.119	0.805	0.286	0.227	0.393	0.004^**^
*Maxillary canine*										
Palatal-ABT-S1 (mm)	1.495	0.422	0.261	0.182	0.590	0.387	< 0.001^***^	< 0.001^***^	0.001^**^	< 0.001^***^
Palatal-ABT-S2 (mm)	2.223	0.623	0.881	0.714	1.201	0.541	< 0.001^***^	< 0.001^***^	0.003^**^	< 0.001^***^
Palatal-ABT-S3 (mm)	3.028	0.789	1.706	1.013	1.975	0.747	< 0.001^***^	< 0.001^***^	0.009^**^	< 0.001^***^
Palatal-ABT-S4 (mm)	8.462	2.413	6.693	2.614	6.938	2.367	< 0.001^***^	< 0.001^***^	0.036^*^	< 0.001^***^
Labial-ABT-S1 (mm)	0.994	0.418	1.361	0.553	1.165	0.494	< 0.001^***^	< 0.001^***^	0.009^**^	0.005^**^
Labial-ABT-S2 (mm)	0.797	0.418	1.129	0.384	0.902	0.407	< 0.001^***^	< 0.001^***^	< 0.001^***^	0.112
Labial-ABT-S3 (mm)	0.511	0.369	0.913	0.443	0.633	0.380	0.001^**^	0.002^**^	0.001^**^	0.135
Labial-ABT-S4 (mm)	1.424	0.698	2.813	1.662	2.125	1.423	< 0.001^***^	< 0.001^***^	< 0.001^***^	0.007^**^
Palatal-ABH (mm)	1.225	0.464	5.790	1.654	3.164	1.477	< 0.001^***^	< 0.001^***^	< 0.001^***^	0.001^**^
Labial-ABH (mm)	2.149	0.923	2.257	0.854	2.319	0.838	0.265	0.349	0.458	0.149

^*^P < 0.05; ^**^ *P* < 0.01; ^***^ *P* < 0.001

As shown in Table 4, after orthodontic treatment, the retractions of the maxillary central and lateral incisor were 5.120 ± 2.545 mm and 5.017 ± 1.691 at the incisal edge level, and 2.277 ± 1.845 mm and 1.797 ± 1.630 mm at the root apex level, respectively. During the retention period, the maxillary incisors showed significant labial movement with a relative intrusion. For the maxillary central incisor, the labial movement was 0.271 ± 0.466 mm at the incisal edge and 0.749 ± 0.564 mm at the root apex. Similarly, the labial movement of the maxillary lateral incisor was 0.150 ± 0.460 mm at the incisal edge and 0.528 ± 0.391 mm at the root apex. Vertically, the incisal edge was relatively intruded (maxillary central incisor: 0.457 ± 0.614 mm; maxillary lateral incisor: 0.240 ± 0.481 mm). As for the maxillary canine, the root apex was displaced mesially (0.738 ± 0.574 mm) during the retention period.

**Table 4:** The teeth movement of maxillary anterior teeth in PBD group

	T2–T1			T3–T2		
	Mean	SD	*p*	*p*	Mean	SD
*Maxillary central incisor*						
Incisal edge-sagittal (mm)	− 5.120	2.545	< 0.001^***^	0.271	0.466	0.006^**^
Incisal edge-vertical (mm)	− 0.300	1.248	0.164	− 0.457	0.614	< 0.001^***^
Root apex-sagittal (mm)	− 2.277	1.845	< 0.001^***^	0.749	0.564	< 0.001^***^
Root apex-vertical (mm)	− 1.517	1.644	< 0.001^***^	− 0.240	0.481	0.002^**^
*Maxillary lateral incisor*						
Incisal edge-sagittal (mm)	− 5.017	1.691	< 0.001^***^	0.150	0.460	0.028^*^
Incisal edge-vertical (mm)	− 0.136	1.215	0.506	− 0.417	0.405	< 0.001^***^
Root apex-sagittal (mm)	− 1.797	1.630	< 0.001^***^	0.528	0.391	< 0.001^***^
Root apex- vertical (mm)	− 1.275	1.246	< 0.001^***^	− 0.186	0.486	0.058
*Maxillary canine*						
Cusp tip-sagittal (mm)	− 2.454	2.460	< 0.001^***^	0.175	0.557	0.137
Cusp tip-transversal (mm)	0.813	1.338	0.007^**^	− 0.117	0.459	0.226
Cusp tip-vertical (mm)	0.067	1.330	0.808	0.050	0.737	0.743
Root apex-sagittal (mm)	− 2.788	1.530	< 0.001^***^	0.738	0.574	< 0.001^***^
Root apex-transversal (mm)	1.000	1.390	0.002^**^	− 0.104	0.420	0.236
Root apex-vertical (mm)	− 0.208	1.537	0.478	0.138	0.571	0.609

Positive values indicate labial, extrusive and buccal teeth movement. Negative values indicate lingual, intrusive and palatal teeth movement

^*^P < 0.05; ^**^ *P* < 0.01; ^***^ *P* < 0.001

### Bone remodeling and tooth movement of maxillary anterior teeth in the LBF group

There were 35 maxillary anterior teeth with LBF at T2 (5 maxillary central incisors, 10 maxillary lateral incisors, and 20 maxillary canines) in the LBF group. As shown in Table 5, the labial ABT of the maxillary central incisor at the S2 and S4 levels showed a significant decrease during the retraction period and a significant increase during the retention period (S2: 1.197 ± 0.653 mm at T1, 0.823 ± 0.660 mm at T2, 1.045 ± 0.532 mm at T3; S4: 1.125 ± 0.332 mm at T1, 0.791 ± 0.627 mm at T2, 1.164 ± 0.651 mm at T3), indicating a recovery of bone fenestration. The palatal ABT of maxillary anterior teeth remained stable during the retention period. The palatal ABH of the maxillary lateral incisor and canine significantly increased after orthodontic treatment and decreased after at least 1 year of retention. The labial ABH of all maxillary anterior teeth were stable during and after orthodontic treatment.

**Table 5:** The alveolar bone thickness and height of maxillary anterior teeth in LBF group

	T1		T2		T3		Overall	T1 vs T2	T2 vs T3	T1 vs T3
	Mean	SD	Mean	SD	Mean	SD	*P*	*p*	*p*	*p*
*Maxillary central incisor*										
Palatal-ABT-S1 (mm)	1.753	0.206	1.788	0.565	1.689	0.371	0.923	0.905	0.699	0.794
Palatal-ABT-S2 (mm)	2.319	0.432	3.493	0.549	3.170	0.822	0.447	0.387	0.685	0.326
Palatal-ABT-S3 (mm)	3.254	0.727	5.669	1.030	4.819	1.208	0.010^*^	0.011^*^	0.278	0.068
Palatal-ABT-S4 (mm)	8.290	1.667	10.622	1.280	10.146	1.681	0.252	0.249	0.198	0.358
Labial-ABT-S1 (mm)	1.360	0.566	1.054	0.425	1.128	0.312	0.001^**^	0.005^**^	0.076	0.010^*^
Labial-ABT-S2 (mm)	1.197	0.653	0.823	0.660	1.045	0.532	0.015^*^	0.049^*^	0.027^*^	0.265
Labial-ABT-S3 (mm)	0.884	0.507	0.335	0.251	0.736	0.334	0.015^*^	0.045^*^	0.412	0.027^*^
Labial-ABT-S4 (mm)	1.125	0.332	0.791	0.627	1.164	0.651	0.186	0.193	0.029^*^	0.884
Palatal-ABH (mm)	0.872	0.126	2.416	1.193	1.235	0.314	0.024^*^	0.051	0.098	0.051
Labial-ABH (mm)	1.612	0.588	1.647	0.939	1.785	0.740	0.674	0.874	0.500	0.453
*Maxillary lateral incisor*										
Palatal-ABT-S1 (mm)	1.569	0.305	0.788	0.640	0.923	0.471	0.003^**^	0.006^**^	0.214	0.003^**^
Palatal-ABT-S2 (mm)	2.014	0.571	1.583	1.155	1.645	0.756	0.355	0.663	0.403	0.105
Palatal-ABT-S3 (mm)	2.562	0.758	2.495	1.688	2.440	1.259	0.243	0.254	0.705	0.159
Palatal-ABT-S4 (mm)	5.680	2.071	5.671	2.241	5.659	2.003	0.746	0.535	0.754	0.609
Labial-ABT-S1 (mm)	0.660	0.312	0.702	0.438	0.789	0.258	0.838	0.894	0.724	0.757
Labial-ABT-S2 (mm)	0.296	0.268	0.351	0.263	0.330	0.201	0.022^*^	0.058	0.476	0.007^**^
Labial-ABT-S3 (mm)	0.151	0.113	0.238	0.122	0.272	0.116	0.987	0.991	0.952	0.977
Labial-ABT-S4 (mm)	1.338	0.451	1.037	0.535	1.248	0.489	0.368	0.248	0.220	0.700
Palatal-ABH (mm)	1.011	0.380	3.671	3.155	1.292	0.623	0.026^*^	0.031^*^	0.020^*^	0.271
Labial-ABH (mm)	1.778	0.572	1.879	0.964	2.086	0.877	0.236	0.655	0.175	0.095
*Maxillary canine*										
Palatal-ABT-S1 (mm)	1.596	0.485	0.980	0.704	1.014	0.516	< 0.001^***^	0.002^**^	0.752	< 0.001^***^
Palatal-ABT-S2 (mm)	2.465	0.694	1.907	1.050	1.801	0.851	0.306	0.242	0.490	0.261
Palatal-ABT-S3 (mm)	3.334	0.827	3.025	1.114	2.988	1.047	0.009^**^	0.031^*^	0.273	0.002^**^
Palatal-ABT-S4 (mm)	9.343	1.479	9.513	2.150	9.477	2.100	0.779	0.832	0.600	0.487
Labial-ABT-S1 (mm)	0.974	0.406	1.080	0.446	1.037	0.399	0.169	0.199	0.729	0.119
Labial-ABT-S2 (mm)	0.778	0.458	0.760	0.390	0.727	0.378	0.800	0.848	0.643	0.448
Labial-ABT-S3 (mm)	0.507	0.411	0.490	0.326	0.459	0.311	0.726	0.628	0.791	0.726
Labial-ABT-S4 (mm)	1.123	0.679	1.574	1.140	1.452	1.271	0.065	0.028^*^	0.099	0.153
Palatal-ABH (mm)	1.151	0.794	3.452	1.972	2.159	1.424	< 0.001^***^	< 0.001^***^	0.002^**^	0.018^*^
Labial-ABH (mm)	2.148	1.069	2.199	1.060	2.310	0.911	0.418	0.724	0.296	0.208

^*^P < 0.05; ^**^ *P* < 0.01; ^***^ *P* < 0.001

As shown in Table 6, after orthodontic treatment, the maxillary incisors had retracted significantly at the incisal edge level (maxillary central incisor: 6.260 ± 1.932 mm; maxillary lateral incisor: 5.580 ± 1.691 mm). The root of the maxillary incisor was less retracted (maxillary central incisor: 0.020 ± 1.674 mm; maxillary lateral incisor: 0.700 ± 1.534 mm), indicating that the maxillary incisors were lingually inclined. During the retention period, the maxillary central incisor showed significant labial movement (incisal edge: 0.740 ± 0.439 mm; root apex: 0.180 ± 0.460 mm) with relative intrusion (incisal edge: 0.400 ± 0.173 mm). Similarly, the labial movements of the maxillary lateral incisor were 0.250 ± 0.280 mm at the incisal edge level and 0.290 ± 0.303 mm at the root apex level. As for the maxillary canine, the root apex moved buccally (1.525 ± 1.655 mm) during orthodontic treatment and moved palatally (–0.215 ± 0.461 mm) during the retention period.

**Table 6:** The teeth movement of maxillary anterior teeth in LBF group

	T2–T1			T3–T2		
	Mean	SD	*p*	Mean	SD	*p*
*Maxillary central incisor*						
Incisal edge-sagittal (mm)	− 6.260	1.932	0.002^**^	0.740	0.439	0.020^*^
Incisal edge-vertical (mm)	0.360	0.777	0.358	− 0.400	0.173	0.007^**^
Root apex-sagittal (mm)	− 0.020	1.674	0.980	0.180	0.460	0.431
Root apex-vertical (mm)	0.280	1.901	0.758	− 0.380	0.390	0.095
*Maxillary lateral incisor*						
Incisal edge-sagittal (mm)	− 5.580	1.743	< 0.001^***^	0.250	0.280	0.020^*^
Incisal edge-vertical (mm)	0.020	1.110	0.956	− 0.210	0.328	0.074
Root apex-sagittal (mm)	− 0.700	1.534	0.183	0.290	0.303	0.014^*^
Root apex- vertical (mm)	0.210	1.575	0.683	0.080	0.434	0.574
*Maxillary canine*						
Cusp tip-sagittal (mm)	− 5.355	1.067	< 0.001^***^	0.305	0.427	0.005^**^
Cusp tip-transversal (mm)	0.940	0.843	< 0.001^***^	− 0.090	0.578	0.495
Cusp tip-vertical (mm)	0.365	1.135	0.167	− 0.140	0.437	0.168
Root apex-sagittal (mm)	− 1.890	1.790	< 0.001^***^	0.495	0.474	< 0.001^***^
Root apex-transversal (mm)	1.525	1.655	0.001^**^	− 0.215	0.461	0.051
Root apex-vertical (mm)	− 0.310	1.422	0.342	− 0.190	0.422	0.058

Positive values indicate labial, extrusive and buccal teeth movement. Negative values indicate lingual, intrusive and palatal teeth movement

^*^P < 0.05; ^**^ *P* < 0.01; ^***^ *P* < 0.001

## Discussion

The boundary of orthodontic tooth retraction has been the subject of a lengthy debate. Although the consensus is to keep the teeth in the alveolar bone during orthodontic retraction, it is challenging to achieve this treatment goal, especially for patients with convex profile and narrow alveolar bone. Numerous CBCT studies have shown that alveolar bone defects (PBD or LBF) frequently occur after maxillary anterior teeth retraction [9–12]. To our knowledge, no study has examined the long-term bone remodeling of maxillary anterior teeth in adult patients with alveolar bone defects. To assess the periodontal health of such teeth, we first analyzed the three-dimensional changes in maxillary anterior teeth and the surrounding alveolar bone in adult patients during orthodontic treatment and retention.

After retraction of maxillary anterior teeth, the incidence of PBD significantly increased from 0 to 67.36%. Unlike the efficient remodeling of labial alveolar bone that follows the retraction of anterior teeth, palatal alveolar bone does not remodel well. Secondary bone apposition on the palatal side is insufficient, and the original morphology of the cortical bone may represent a boundary for maxillary anterior teeth retraction. Once the anterior teeth contact the cortical bone, bone dehiscence could occur. In a study by Ahn et al. [9], the loss of vertical alveolar bone of the maxillary central incisor on the palatal side was an average of 3.65 mm after retraction treatment. In our study, the vertical bone loss on the palatal side in the PBD group was 5.7 mm in the maxillary central incisor and 7.20 mm in the maxillary lateral incisor, which showed severe bone dehiscence. It should be noted that the incidence and severity of PBD in maxillary anterior teeth after retraction were high because only patients with alveolar bone defects were included. Besides, the alveolar bone change in maxillary anterior teeth during retraction is highly related to the amount and type of tooth movement [20, 21]. The tooth movement of maxillary anterior teeth retraction in the PBD group was close to bodily movement, which might increase the severity of PBD.

**Fig. 2:**
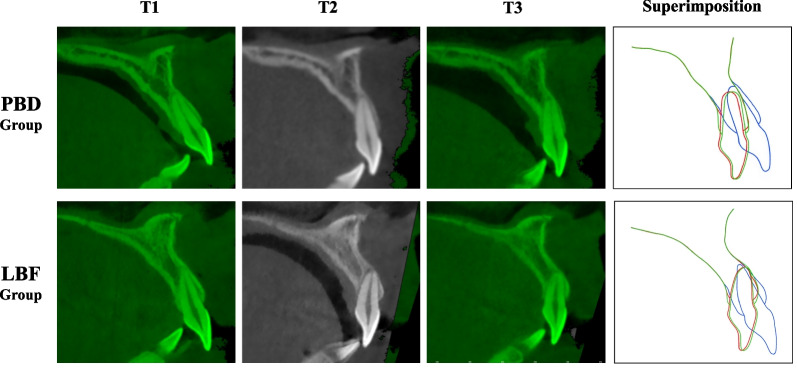
CBCT images and superimposition of maxillary incisor and surrounding alveolar bone in palatal bone dehiscence (PBD) and labial bone fenestration (LBF) groups during the orthodontic treatment and retention period (blue: pre-treatment; red: post-treatment; green: retention)

At present, there is no consensus on the capacity of alveolar bone remodeling during the retention period, particularly for teeth with alveolar bone defects. According to the study by Remmelink et al. [16] and Wainwight et al. [22], severe bone dehiscence, which occurs when the root penetrates the cortical plate, cannot be repaired. Surprisingly, we found that there was a layer of new cortical bone on the palatal side of maxillary anterior teeth. The palatal bone dehiscence was significantly recovered during the retention period. Moreover, the thickness of the palatal bone was also significantly increased. Our results are consistent with a case report that showed that palatal bone dehiscence of maxillary incisors in a 31-year-old woman with four premolar extractions and mini-screw anchorage was recovered 10 years after treatment [23]. Another study reported that the height and thickness of palatal bone at the cervical level were significantly increased in adolescent patients after 18–24 months of retention [15]. To further analyze treatment stability, we superimposed post-treatment and retention CBCT images. We found that the root of the maxillary anterior teeth labially moved, which indicates that the teeth moved into alveolar bone housing. Due to the labial movement of maxillary anterior teeth, the thickness of the labial bone significantly decreased. Our results are consistent with those of Chaison et al. [24], who reported that regeneration of alveolar bone is related to the torque relapse of maxillary anterior teeth during the retention period. The reorientation of maxillary anterior teeth and secondary bone apposition may both contribute to the regeneration of cortical bone during the retention period (Fig. 2). Importantly, although palatal bone was recovered, the bone height and thickness did not fully return to pre-treatment levels.

After maxillary anterior teeth retraction, the incidence of LBF (24.31%) was lower than that of PBD, and bone fenestration was detected mainly in the apical region of the labial root. Different from the approximate bodily movement in the PBD group, the type of maxillary anterior teeth retraction in the LBF group was mainly an uncontrolled tipping movement, which induced LBF in the apical region of the root. As for maxillary canines, the buccal movement of the root increased the occurrence of LBF. Hence, torque control of maxillary anterior teeth is essential during retraction. After at least 1 year of retention, the incidence of LBF had decreased significantly. A continuous bone covering occurred over the exposed root apex (Fig. 2). Our findings are consistent with those of Robert et al. [25], who also reported that LBF was partly repaired. CBCT superimposition revealed that the root of the maxillary canine moved palatally into alveolar bone housing during the retention period. Although the root of maxillary incisors moved labially, bone still covered the labial side. Hence, we speculate that LBF in maxillary incisors is mainly repaired by osteoblastic periodontal remodeling.

Treatment stability is an essential concern in the orthodontic field. Relapse after orthodontic treatment is a trend of returning to its initial position, which is generally inevitable and unpredictable [26]. Quaglio et al. [27] analyzed the relapse of maxillary anterior crowding in extraction patients, and found that the stability of maxillary anterior alignment was 88.12%. The support of the alveolar bone plays an important role in dental relapse [24]. A retrospective study conducted by Rothe et al. [28] found that the decreased cortical thickness could increase the risk of incisor relapse. In our study, we found that maxillary anterior teeth with alveolar bone defects are less stable, and their position significantly changed during the retention period in both PBD and LBF groups. Maxillary anterior teeth spontaneously reoriented, with the root moving into the bone, which partly contributed to bone recovery. It is worth mentioning that there was no significant crowding or spacing relapse in our study, due to good patient compliance with retainer wear. That is to say, the slight relapse of the root could not be prevented by a removal retainer.

Esthetics is a major motivating factor for adult patients with maxillary protrusion seeking orthodontic treatment. To address this issue, maximum maxillary anterior teeth retraction with premolar extraction is always needed. Current evidence suggests that bone remodeling capacity declines with age due to weakened stem cell function [29]. Adult patients have limited bone remodeling capacity, and a high incidence of alveolar bone defects after orthodontic treatment has been reported [9]. It is imperative to evaluate the long-term periodontal safety of maxillary anterior teeth after retraction. We were pleased that our adult patients showed good repair of alveolar bone defects during the retention period. Several factors might contribute to alveolar bone covering in adult patients. First, the included patients are young adults (age: 24.86 ± 4.10 years), who still have a good bone remodeling capacity. Second, unlike mandibular incisors with bone dehiscence and gingival recession, the thick palatal gingival tissue of maxillary anterior teeth might aid alveolar bone regeneration by conserving periodontal ligaments, which participate in alveolar bone remodeling. Despite the fact that bone dehiscence occurred on the palatal side of maxillary incisors, there was no gingival recession or root exposure, due to the coverage of thick palatal gingival tissue.

Our results suggest that maxillary anterior teeth with bone dehiscence and bone fenestration may develop bone covering during the retention period. However, there are several considerations that should be emphasized in the interpretation of our results. First, although CBCT is accurate and reliable for measuring alveolar bone defects, they may still be overestimated [30–32]. In this study, CBCT with 0.25 mm scanning voxel size was used to detect alveolar bone defects. When the bone thickness was less than 0.25 mm, these conditions could not be detected. Second, the alveolar bone defects that occurred in maxillary anterior teeth were primary palatal bone dehiscence and labial bone fenestration; labial bone dehiscence and palatal bone fenestration were not observed in this study. Hence, only the former were analyzed. Finally, although our results demonstrate that alveolar bone defects can be repaired during retention, maximum retraction may not be acceptable for all adult patients. Bone remodeling capacity varies among people. If periodontal inflammation is not controlled during the retention period, anterior teeth with less bone support are vulnerable to further damage. Hence, the amount of maxillary anterior teeth retraction should be based on the pre-treatment anatomic limit, particularly for a patient with maxillary protrusion and a thin alveolar bone. Once the alveolar bone defect occurs after orthodontic treatment, it is still essential to perform a long-term follow-up examination.

## Conclusion

For adult patients with maxillary protrusion, the palatal bone dehiscence and labial bone fenestration of maxillary anterior teeth caused by orthodontic retraction significantly improved during the retention period, indicating good long-term bone remodeling. Our findings suggest that a combination of spontaneous reorientation of maxillary anterior teeth and bone remodeling contributed to alveolar bone covering in these patients.

## Data Availability

The datasets used and/or analyzed during the current study are available from the corresponding author on reasonable request.
